# Using Machine Learning to Predict Early Onset Acute Organ Failure in Critically Ill Intensive Care Unit Patients With Sickle Cell Disease: Retrospective Study

**DOI:** 10.2196/14693

**Published:** 2020-05-13

**Authors:** Akram Mohammed, Pradeep S B Podila, Robert L Davis, Kenneth I Ataga, Jane S Hankins, Rishikesan Kamaleswaran

**Affiliations:** 1 Center for Biomedical Informatics University of Tennessee Health Science Center Memphis, TN United States; 2 Faith and Health Division Methodist Le Bonheur Healthcare Memphis, TN United States; 3 Center for Sickle Cell Disease University of Tennessee Health Science Center Memphis, TN United States; 4 Department of Hematology St Jude Children's Research Hospital Memphis, TN United States; 5 Department of Biomedical Informatics Emory University School of Medicine Atlanta, GA United States

**Keywords:** multiple organ failure, sickle cell disease, machine learning, electronic medical record, hematology

## Abstract

**Background:**

Sickle cell disease (SCD) is a genetic disorder of the red blood cells, resulting in multiple acute and chronic complications, including pain episodes, stroke, and kidney disease. Patients with SCD develop chronic organ dysfunction, which may progress to organ failure during disease exacerbations. Early detection of acute physiological deterioration leading to organ failure is not always attainable. Machine learning techniques that allow for prediction of organ failure may enable early identification and treatment and potentially reduce mortality.

**Objective:**

The aim of this study was to test the hypothesis that machine learning physiomarkers can predict the development of organ dysfunction in a sample of adult patients with SCD admitted to intensive care units (ICUs).

**Methods:**

We applied diverse machine learning methods, statistical methods, and data visualization techniques to develop classification models to distinguish SCD from controls.

**Results:**

We studied 63 sequential SCD patients admitted to ICUs with 163 patient encounters (mean age 30.7 years, SD 9.8 years). A subset of these patient encounters, 22.7% (37/163), met the sequential organ failure assessment criteria. The other 126 SCD patient encounters served as controls. A set of signal processing features (such as fast Fourier transform, energy, and continuous wavelet transform) derived from heart rate, blood pressure, and respiratory rate was identified to distinguish patients with SCD who developed acute physiological deterioration leading to organ failure from patients with SCD who did not meet the criteria. A multilayer perceptron model accurately predicted organ failure up to 6 hours before onset, with an average sensitivity and specificity of 96% and 98%, respectively.

**Conclusions:**

This retrospective study demonstrated the viability of using machine learning to predict acute organ failure among hospitalized adults with SCD. The discovery of salient physiomarkers through machine learning techniques has the potential to further accelerate the development and implementation of innovative care delivery protocols and strategies for medically vulnerable patients.

## Introduction

### Background

Sickle cell disease (SCD), one of the most common genetic disorders, affects millions across the globe [1]. It was the first monogenic disorder to be characterized at the molecular level. It is characterized by the presence of abnormal hemoglobin S, which, under hypoxic conditions, causes sickling of red blood cells, resulting in tissue and organ damage. Among an array of complications afflicting patients with SCD, the most devastating is major organ failure, including pulmonary failure, end-stage renal disease, stroke, and heart failure [1]. A 4-decade observational study reported that, by the fifth decade of life, up to half of all patients with SCD had documented irreversible organ damage [2]. Organ dysfunction may manifest or worsen during hospitalizations, when disease complications arise. Thus, therapy supplemented by predictive analytics can potentially improve the outcomes of patients with SCD [3]. Early diagnosis of acute organ dysfunction may allow for early intervention, thereby preventing or reducing the severity of organ failure, particularly during hospitalization for acute complications.

Early recognition of organ failure may [4,5] thereby enable clinicians to provide targeted therapies to improve outcomes. Various scoring methods have been developed for qualifying organ dysfunction, including Acute Physiology and Chronic Health Evaluation [6], Multi-Organ Dysfunction Score [7], *quick* Sequential Organ Failure Assessment [8] and Sequential Organ Failure Assessment (SOFA) [9]. The SOFA is a mortality prediction score that is based on the degree of dysfunction of six organ systems. The score is calculated at admission and every 24 hours until discharge, using the worst parameters measured during the previous 24 hours. Compared with other scoring methods, the use of SOFA allowed us to retrospectively quantify both the number and severity of individual organ dysfunction.

### Objectives

In this retrospective study, we used serial calculations of SOFA to identify the onset of organ failure and then used physiomarkers in machine learning models to predict organ failure for patients with SCD presenting with a severe, acute painful crisis. Our hypothesis was that physiomarkers [10] identified by machine learning methods can be used to predict organ failure.

## Methods

### Cohort

**Figure 1:**
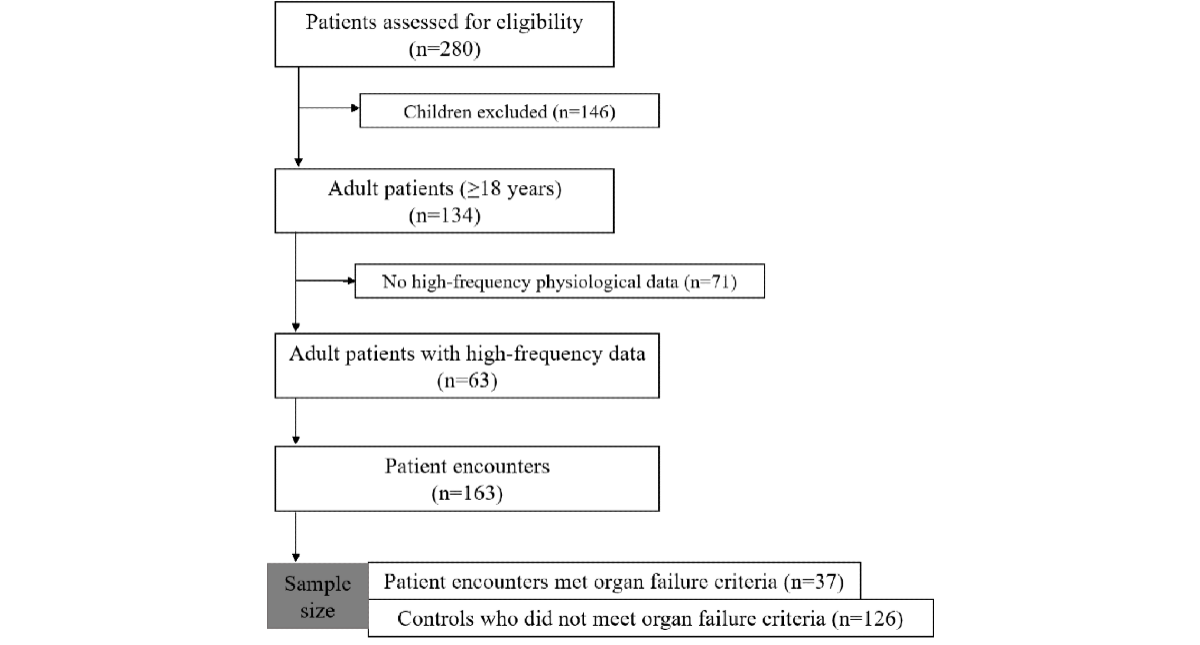
Consolidated Standards for Reporting of Trials diagram describing the study cohort.

Continuous physiologic data were collected on 134 adult subjects with SCD admitted to intensive care units (ICUs) at Methodist Le Bonheur Healthcare hospitals, Memphis, Tennessee, United States, between June 2017 and March 2018. Patients were retrospectively identified using a discharge International Classification of Diseases, Tenth Revision (ICD-10) code of D57.*. Of the 134 unique patients, 71 patients who did not have at least 24 hours of continuous physiologic data were excluded from the analysis. We studied patients who had at least 24 hours of continuous high-frequency physiologic data available before the time of organ failure onset (identified using SOFA criteria). A total of 63 unique adult subjects who had SCD (discharge ICD-10 code of D57.*) and were admitted to the medical, surgical, neurological, and cardiac ICUs and had continuous physiologic data available were retrospectively identified and included in the study. These 63 patients had 163 encounters (Figure 1). Of the 163 encounters, 37 patient encounters corresponding to 29 unique patients met organ failure criteria. The inclusion and exclusion criteria are summarized in Figure 1. Demographic and clinical data on cases and controls were collected from the electronic medical record (EMR) using Cerner’s Web Intelligence reporting module (Cerner Health Facts). Each patient admission was considered as a separate patient encounter if the interval between admissions was at least for 1 month. The principal or admit diagnosis was identified using the ICD-10 codes. Patients with organ failure at admission were excluded from the analysis.

High-frequency physiologic data were collected at the frequency of once per minute from the time of admission until discharge. A total of 5 physiologic characteristics were used in the analysis, including heart rate (HR), respiratory rate (RR), systolic blood pressure (SBP), diastolic blood pressure (DBP), and mean blood pressure (MBP). These vital signs were selected because they were always obtained in all patients admitted to ICUs, and these can be used in identifying organ failure using a minimal set of physiologic data. The main outcome of organ failure was the failure of at least one organ or system (cardiovascular, liver, respiratory, coagulation, central nervous system, or renal), and cases were defined as patients meeting an increase in a serially calculated SOFA criteria by at least one score within a 24-hour rolling window, from admission till discharge. Event time (t_onset_) was recorded as the earliest time stamp of every occurrence of organ failure, as defined independently by SOFA for each patient. Patients without 24 hours of physiological data before the t_onset_ (for cases and controls) were excluded. To normalize our prediction time horizon, we created relative alignments of time windows, pivoted to t_onset_. This study was approved by The University of Tennessee Health Science Center and Methodist Le Bonheur Hospital Institutional Review Boards, and it was performed in compliance with the ethical principles for medical research involving human subjects from the Declaration of Helsinki.

### Feature Extraction and Feature Selection

We utilized Python libraries for extracting features from each of the physiological data streams, including a combination of temporal, frequency, and statistical features (Multimedia Appendix 1) [11]. Features were derived from six overlapping 3-hour time intervals, with a stride of 1 hour, from 1 to 4 hours to 6 to 9 hours before organ failure, so that we could build predictive models at different times before organ failure. For controls (patients with SCD not developing organ failure), we extracted these same features from 3-hour intervals before a random time period (identified by time stamp) during their ICU stay. The detailed feature extraction for organ failure cases and controls is shown in Multimedia Appendix 2. We performed feature selection using multiple null hypothesis testing, using the Benjamini-Yekutieli procedure and Mann-Whitney *U* test. Finally, we ranked the features using random forest (RF) [12] feature importance algorithm and applied various feature thresholds to select subsets of features that were most discriminatory among cases and controls.

### Machine Learning Algorithms

Multilayer perceptron (MLP), support vector machine (SVM), RF, and logistic regression (LR) methods were used for building classification models. These methods were adopted because of their successful applications to medical datasets for disease classification [13-18]. 

An MLP is a deep, feed-forward neural network comprising an input layer, an output layer, and at least two or more hidden layers [19]. MLP has been used in a variety of applications, including electroencephalogram signal classification [15], heart disease diagnosis [20], ovarian tumor classification [21], and continuous speech recognition [22]. The MLP architecture used in this study comprised five hidden layers of 512, 256, 128, 64, and 16 neurons. We applied batch normalization [23] before activation, using the rectified linear unit. To avoid overfitting, we further imposed a dropout [24] ratio of 0.3. The output layer performed binary classification using the sigmoid activation, and our loss function used the Adam optimizer.

The SVM is a multivariate machine learning approach for classifying samples through a pattern recognition analysis [25]. SVM aims to find the best hyperplane that separates all data points of one class compared with those of another class. We used a radial basis function as the kernel parameter for model building.

The RF classifier is well suited to the classification of medical data because of the following advantages: (1) it performs embedded feature selection, (2) it incorporates interactions between predictors, (3) it allows the algorithm to learn both simple and complex classification functions accurately, and (4) it is applicable to both binary and multicategory classification tasks [12]. On the basis of the out-of-bag error [26], we identified 500 trees in the RF models as the optimal number of trees.

LR can be used as a machine learning method used to predict the value of a binary variable based on its relationship with predictor variables [27]. The *P* value for statistical testing of variable significance for *inclusion-in* and *exclusion-from* the model was set to *P*=.05 and *P*=.10, respectively, and LIBLINEAR solver was used for the optimization function.

### Statistical Analysis and Machine Learning Framework

Python scikit-learn machine learning library [28] was used for calculating descriptive statistical measures, for feature selection, and for building machine learning classifiers. Bivariate LR, bootstrap, and Bayesian bootstrap (adjusted for weights) were used to assess the predictability of the features generated for predicting organ failure [29]. We used nonparametric Kruskal-Wallis statistical tests to analyze the difference among the five physiological signals (DBP, SBP, MBP, HR, and RR). Each of these five signals had six feature measurements, namely, mean, energy ratio, fast Fourier transform, linear trend, quantile, and continuous wavelet transform.

### Cross Validation

Models were developed from the distinct time intervals and tested on patients who were not included in the training of the model. For model selection and accuracy estimation, we used 5-fold cross validation [30]. This technique divides data into five equal and discrete folds and uses four folds for model generation, whereas predictions are generated and evaluated using the remaining single fold. This step is subsequently repeated five times, so each fold is tested against the other four folds. We further ran each of these 5-fold cross-validation models 10 times by shuffling the data in each iteration and averaged the performance metrics from all iterations to reduce bias.

## Results

### Patient Characteristics

Tables 1 and 2 outline the descriptive-level characteristics of demographics and clinical characteristics of 163 encounters. Four patients with organ failure at admission were omitted from further analysis and their data are not shown in Table 1.

**Table 1:** Encounter-level demographics and principal diagnosis of patients in the overall cohort (n=163).

Variable^a^			Total cohort		Organ failure (yes)		Organ failure (no)		*P* value
Total sample, n (%)			163 (100.0)		37 (22.7)		126 (77.3)		N/A^b^
Age (years), mean (SD)			30.7 (9.8)		35.2 (12.9)		29.3 (8.3)		.01^c^
Female, n (%)			87 (53.4)		24 (64.9)		63 (50.0)		.11
African American, n (%)			163 (100.0)		37 (100.0)		126 (100.0)		N/A
**Admit diagnosis, n (%)^d^**									
	Vaso-occlusive event (pain or acute chest syndrome)	130 (79.8)		23 (62.2)		107 (84.9)		.003^c^	
	Nonvaso-occlusive crises pain	7 (4.3)		4 (10.8)		3 (2.4)		.05^c^	
	Infection/sepsis	7 (4.3)		2 (5.4)		5 (4.0)		.66	
	Other^e^	19 (11.6)		8 (21.6)		11 (8.7)		.03^c^	

^a^For continuous variables, independent *t* test was used; for categorical variables, Chi-square test of independence was used. Fisher exact test was used for variables with cell counts of less than 5.

^b^N/A: Not applicable.

^c^Statistically significant at *P*=.05.

^d^The admit diagnoses are based on the International Classification of Diseases, Tenth Revision, Clinical Modification codes at the admission time.

^e^Other category includes respiratory distress, sickle cell disease without crisis, diabetes complications (diabetic ketoacidosis/hyperglycemia), pneumonia, myocardial infarction, hematemesis, cough, and deep venous thrombosis.

The mean age of the patient encounters in the cohort was 30.7 years (9.8 years); all patients were African American, and there were more females, 53.4% (87/163), than males, 46.6% (76/163). Admit diagnoses of vaso-occlusive event (pain or acute chest syndrome; 130/163, 79.8%), nonvaso-occlusive crises pain (7/163, 4.3%), and infection/sepsis (7/163, 4.3%) were common (Table 1). Both vaso-occlusive and nonvaso-occlusive events significantly altered between the patient with *organ failure* and *no organ failure* groups. Patients with organ failure had longer hospital stays (3.2 additional hospital days; *P*=.03) than controls, and they had higher severity of illness (*P*<.001) and risk of mortality (*P*<.001; Table 2).

**Table 2:** Encounter-level clinical characteristics of patients in the overall cohort (n=163).

Variable^a^		Total cohort	Organ failure (yes)	Organ failure (no)	*P* value	
Encounters, n (%)		163 (100.0)	37 (22.7)	126 (77.3)	—^b^	
Encounter through emergency department, n (%)		134 (82.2)	33 (89.2)	101 (80.2)	.33	
Length of stay (days), mean (SD)		5.3 (4.7)	7.7 (8.4)	4.5 (2.5)	.03^c^	
**APR-DRG^d^ severity of illness, n (%)**						**<.001**
	Minor	52 (37.1)	7 (19.4)	45 (43.2)		
	Moderate	40 (28.6)	7 (19.4)	33 (31.7)		
	Major	40 (28.6)	15 (41.7)	25 (24.0)		
	Extreme	8 (5.7)	7 (19.4)	1 (1.0)		
**APR-DRG risk of mortality, n (%)**						**<.001**
	Minor	93 (66.4)	14 (38.9)	79 (76.0)		
	Moderate	26 (18.6)	6 (16.7)	20 (19.2)		
	Major	12 (8.6)	8 (22.2)	4 (3.9)		
	Extreme	9 (6.4)	8 (22.2)	1 (1.0)		
**Discharge disposition, n (%)**						**<.001**
	Home	147 (90.2)	24 (64.9)	123 (97.6)		
	Hospice or home health services	7 (4.3)	5 (13.5)	2 (1.6)		
	Expired	5 (3.1)	5 (13.5)	0 (0.0)		
	Other	4 (2.5)	3 (8.1)	1 (0.8)		

^a^For continuous variables, independent *t* test was used; for categorical variables, Chi-square test of independence was used. Fisher exact test was used for variables with cell counts of less than 5.

^b^Not available.

^c^*P*<.05.

^d^APR-DRG: all patient refined-diagnosis related group.

### Feature Selection

Feature selection was performed to reduce the number of features, and the reduced feature set was fed into each of the classifiers. The sample distribution for each of the six datasets for 3-hour observational periods is given in Table 3. The number of patients varies with the availability of data during each time window.

**Table 3:** Sample distribution and number of features for each dataset using organ failure.

Interval before organ failure onset (hours)	Organ failure events, n	Control events, n
6-9	27	97
5-8	22	90
4-7	22	89
3-6	29	83
2-5	29	88
1-4	29	79

### Model Performance

**Figure 2:**
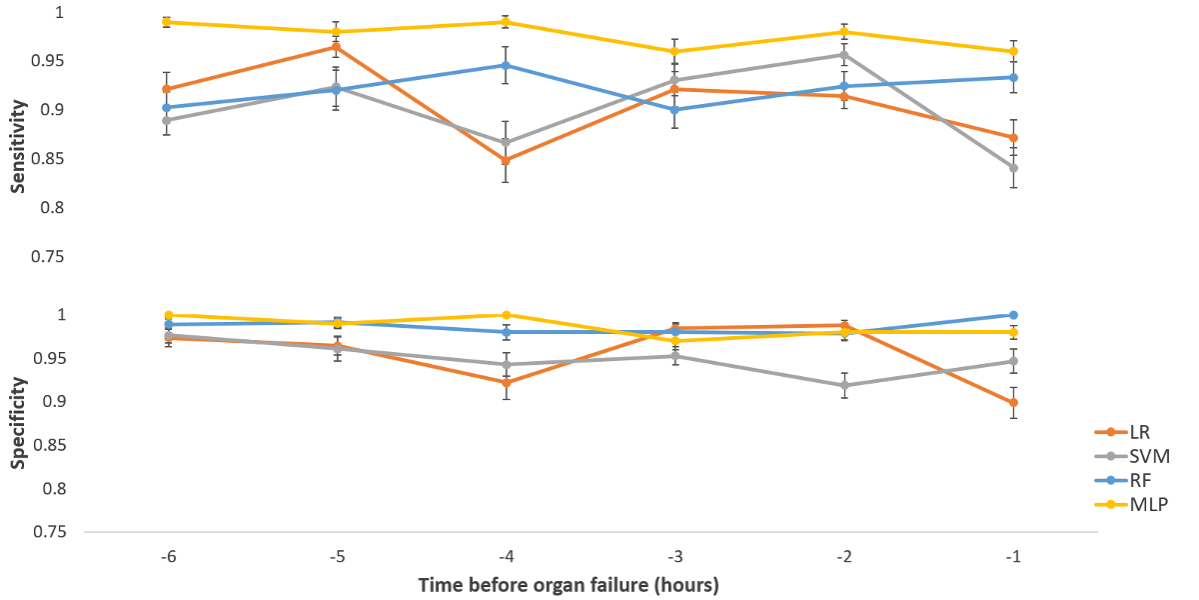
Average sensitivity, specificity for support vector machine, random forest, logistic regression, multilayer perceptron, and sickle cell disease models using each of the six 3-hour datasets. LR: logistic regression; SVM: support vector machine; RF: random forest; MLP: multilayer perceptron.

The average sensitivity and specificity from all models for each of the six time periods are given in Figure 2. The MLP model achieved an average sensitivity and specificity of 96% and 98%, respectively, an hour before organ failure (Figure 2 and Multimedia Appendix 3). Among the four classifiers, MLP performed better than SVM, LR, and RF in predicting SCD with organ failure.

**Figure 3:**
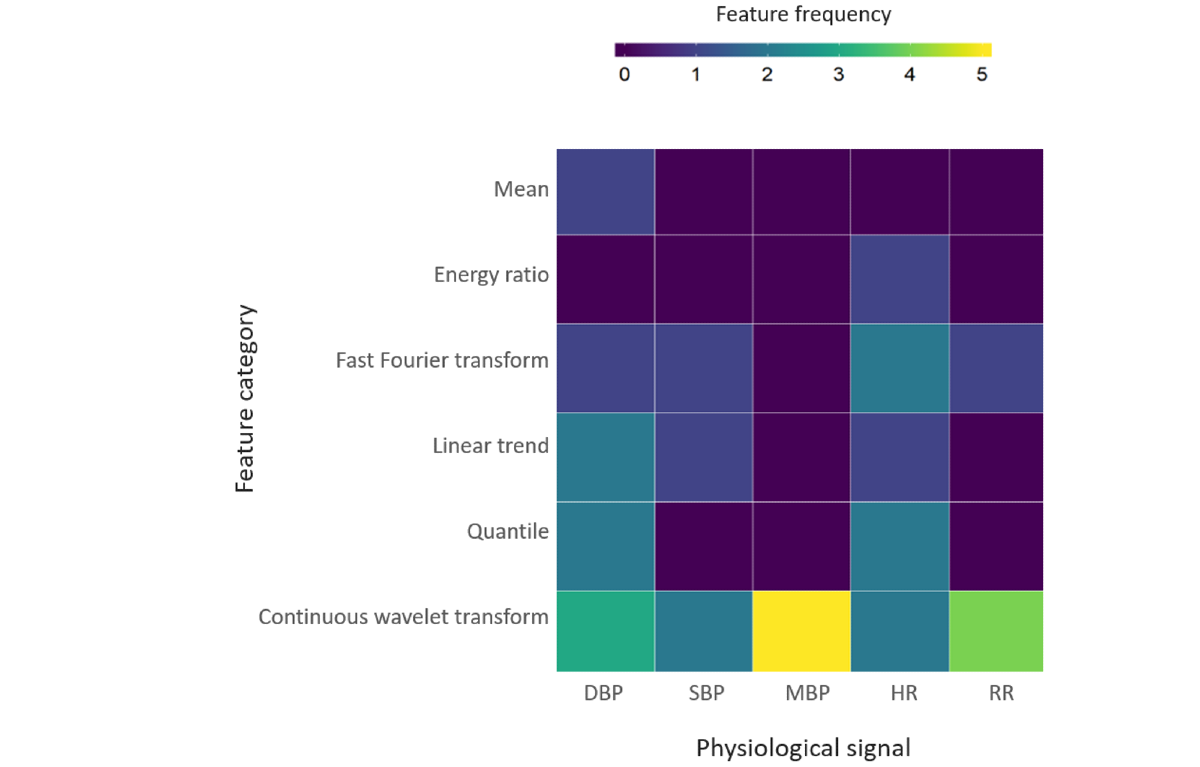
Features derived from physiologic signals up to six hours before organ failure. DBP: diastolic blood pressure; SBP: systolic blood pressure; MBP: mean blood pressure; HR: heart rate; RR: respiratory rate.

RF classifier identified the continuous wavelet transform generated from MBP time series physiologic variable as the most important feature, followed by continuous wavelet transform feature generated from respiratory rate of the time series data. Figure 3 shows the frequency of the top 30 important features generated from the five physiologic signals. Each box in the heat map represents the frequency of a feature (y-axis) generated from the physiological signal (x-axis). The dark purple color in Figure 3 represents the absence of the feature, whereas the dark yellow color represents the most frequently present feature. The list of features ranked by their importance is given in Multimedia Appendix 4. The description of each feature is presented in Multimedia Appendix 1. The Kruskal-Wallis statistical test found no difference among the features extracted from physiological signals (H statistic=5.029; *P*=.28, possibly reflecting the relatively small sample size.

## Discussion

### Principal Findings

Acute organ failure is a major challenge in people with SCD, especially among adults experiencing an acute disease complication. The ability of predictive algorithms to identify patients at high risk for organ deterioration by using routinely collected physiological data can provide important early warnings of impending physiological deterioration. Such information can aid clinical decision making and may even eventually be useful in guiding early goal-directed therapy. In this retrospective study, we demonstrated that the machine learning–based prediction models could accurately distinguish patients with SCD at risk for developing organ failure up to 6 hours before the onset. The classifiers and the selected physiologic features may facilitate accurate, unbiased SCD diagnosis and effective treatment, ultimately improving prognosis.

The selection of relevant features involved in SCD with organ failure remains a challenge [31,32]. Therefore, we wanted to find a subset of physiologic features that are sufficiently informative to distinguish between patients with SCD at risk of developing organ failure and those who are not at risk. To extract useful information from continuous physiologic data of patients with SCD and to reduce dimensionality, feature-selection algorithms were systematically investigated. As we have demonstrated in the results, selecting smaller subsets of features allowed for the high performance of our classification models. Salient physiomarkers (such as fast Fourier transform, energy, and continuous wavelet transform) derived from the physiological signals, such as blood pressure, HR, and RR, may precede acute organ failure in patients with SCD, as suggested by the results in this study. A shortcoming of machine learning is that these physiomarkers are neither observable by physicians nor readily interpretable; instead, their benefit is primarily toward the early prediction of impending physiologic deterioration, as well as alerting health care providers of that fact. Further research is needed to understand how to use these alerts to guide the personalized care of patients with SCD.

The high-frequency data were captured at 1-min intervals, and we only studied patients who had at least 24 hours of continuous high-frequency physiologic data available before the time of organ failure onset (identified using SOFA criteria). Patients admitted to the ICU with organ failure were excluded, as were patients without a full 24 hour of preceding data. It is also possible that some patients who were too sick may not have been connected to the monitors, which may have introduced the selection bias. There is a need for future research to focus on developing models that rely on less data before organ failure.

Other limitations are also important to mention. First, we developed the machine learning model on a small subset of patients, specific to the Mid-South of the United States, potentially reducing generalizability. Moreover, the data were highly imbalanced, with more non–organ failure cases compared with organ failure cases, making data-driven approaches difficult to implement. Missing data elements identified in the data were a major hindrance for model validation; thus, these may have contributed to poor validation in some of the cross-validation folds. Although we included admission/encounter in the machine learning model building if the intervals between admissions were at least 1 month, a patient may be more likely to have organ failure in the subsequent encounter. With larger patient data, in the future, we can restrict events to a single event per patient.

For the purposes of this particular study, the SOFA scores were used only to classify cases and controls and to determine the time of organ failure onset. The machine learning models to distinguish cases and controls were built using a limited set of continuously streaming physiological data. There is an inherent difference in how SOFA data are collected and used versus how data for machine learning were collected and used. Moreover, the time of SOFA scores is inherently delayed, to some unknown degree, and this leads to noise in any predictive model whose goal is dependent on the timing of an event (such as organ failure). As data to compute SOFA scores may be delayed in being entered into the EMR, our proposed machine learning model could be used as an alternative for timely diagnosis. Therefore, additional data are required to develop a more robust and generalizable model.

### Conclusions

In conclusion, we showed, as a proof of principle, that machine learning can accurately predict the development of organ failure in ICU patients with SCD up to 6 hours before onset. This finding is significant because it may optimize the early recognition of serious disease complications and allow for the implementation of early interventions. As future plans, we would like to extend this study to develop a multiclass machine learning classification model to predict the type of organ failure from each of the six organ systems as we collect sufficient data from each organ system.

## Appendix

Multimedia Appendix 1 Temporal, frequency, and statistical features.

Multimedia Appendix 2 Illustration of feature extraction for cases and controls. Each dashed box represent 3-hour physiological data. For cases, the green and blue arrow represent data from one and two-hour prior to organ failure onset, respectively. For controls, gray arrows represent a random 3-hour physiological data.

Multimedia Appendix 3 Average sensitivity, specificity for multi-layer perceptron (MLP), support vector machine (SVM), random forest (RF), and logistic regression (LR).

Multimedia Appendix 4 The list of features ranked by their importance.

## Abbreviations

DBP: diastolic blood pressure
EMR: electronic medical record
HR: heart rate
ICU: intensive care unit
LR: logistic regression
MBP: mean blood pressure
MLP: multilayer perceptron
RF: random forest
RR: respiratory rate
SBP: systolic blood pressure
SCD: sickle cell disease
SOFA: Sequential Organ Failure Assessment
SVM: support vector machine
t_onset_: event time
