# Supplementary figures and images for: Using Machine Learning to Predict Early Onset Acute Organ Failure in Critically Ill Intensive Care Unit Patients With Sickle Cell Disease: Retrospective Study
Source: J Med Internet Res. 2020 May 13;22(5):e14693. doi: 10.2196/14693 (PMC7254279; doi:10.2196/14693)

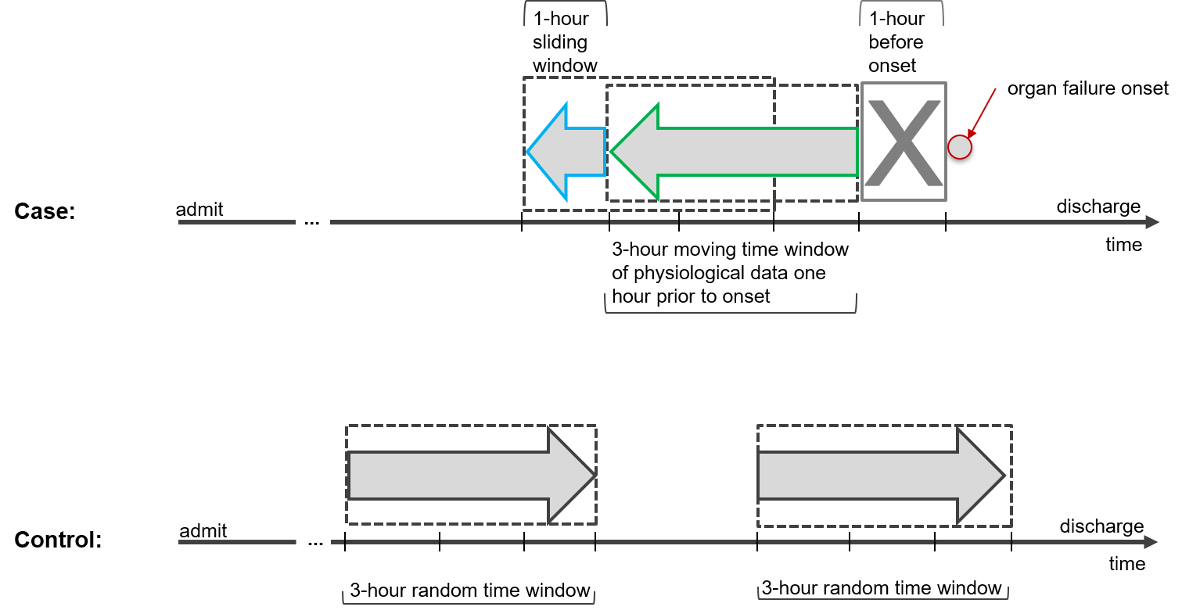

Supplement: Multimedia Appendix 2 [file jmir_v22i5e14693_app2.png]
